# Cardiovascular risk factors as determinants of retinal and skin microvascular function: The Maastricht Study

**DOI:** 10.1371/journal.pone.0187324

**Published:** 2017-10-27

**Authors:** Ben M. Sörensen, Alfons J. H. M. Houben, Tos T. J. M. Berendschot, Jan S. A. G. Schouten, Abraham A. Kroon, Carla J. H. van der Kallen, Ronald M. A. Henry, Annemarie Koster, Pieter C. Dagnelie, Nicolaas C. Schaper, Miranda T. Schram, Coen D. A. Stehouwer

**Affiliations:** 1 CARIM School for Cardiovascular Diseases, Maastricht University, Maastricht, the Netherlands; 2 Department of Internal Medicine, Maastricht University Medical Center+, Maastricht, the Netherlands; 3 University Eye Clinic Maastricht, Maastricht University Medical Center+, Maastricht, the Netherlands; 4 Heart and Vascular Center, Maastricht University Medical Center+, Maastricht, the Netherlands; 5 CAPHRI Care and Public Health Research Institute, Maastricht University, Maastricht, the Netherlands; 6 Department of Social Medicine, Maastricht University, Maastricht, the Netherlands; 7 Department of Epidemiology, Maastricht University, Maastricht, the Netherlands; University of Illinois at Urbana-Champaign, UNITED STATES

## Abstract

**Objective:**

*Microvascular* dysfunction is an important underlying mechanism of *microvascular* diseases. Determinants (age, sex, hypertension, dyslipidemia, hyperglycemia, obesity, and smoking) of *macrovascular* diseases affect large-artery endothelial function. These risk factors also associate with microvascular diseases. We hypothesized that they are also determinants of microvascular (endothelial) function.

**Methods:**

In The Maastricht Study, a type 2 diabetes-enriched population-based cohort study (n = 1991, 51% men, aged 59.7±8.2 years), we determined microvascular function as flicker light-induced retinal arteriolar %-dilation and heat-induced skin %-hyperemia. Multiple linear regression analyses were used to assess the associations of cardiovascular risk factors (age, sex, waist circumference, total-to-high-density lipoprotein (HDL) cholesterol ratio, fasting plasma glucose (FPG), 24-h systolic blood pressure, and cigarette smoking) with retinal and skin microvascular function.

**Results:**

In multivariate analyses, age and FPG were inversely associated with retinal and skin microvascular function (regression coefficients per standard deviation (SD) were -0.11SD (95%CI: -0.15;-0.06) and -0.12SD (-0.17;-0.07) for retinal arteriolar %-dilation and -0.10SD (-0.16;-0.05) and -0.11SD (-0.17;-0.06) for skin %-hyperemia, respectively. Men and current smokers had -0.43SD (-0.58;-0.27) and -0.32SD (-0.49;-0.15) lower skin %-hyperemia, respectively. 24-h systolic blood pressure, waist circumference, and total-to-HDL cholesterol ratio were not statistically significantly associated with these microvascular functions.

**Conclusions:**

Associations between cardiovascular risk factors and retinal and skin microvascular function show a pattern that is partly similar to the associations between cardiovascular risk factors and macrovascular function. Impairment of microvascular function may constitute a pathway through which an adverse cardiovascular risk factor pattern may increase risk of diseases that are partly or wholly of microvascular origin.

## Introduction

Microvascular dysfunction is an important underlying mechanism in common diseases that are partly or wholly of *microvascular* origin such as heart failure [1], (lacunar) stroke [2], depression [3], cognitive decline [4], retinopathy [5], chronic kidney disease [6], and neuropathy [5].

However, determinants of microvascular dysfunction in the general population are mostly unknown. Although several studies [7–13] have investigated potential determinants of microvascular function, these studies were conducted in small numbers of highly selected individuals [7–13] and were insufficiently adjusted [7–13] for potential confounders, which limits translation to the general population.

In the general population, age, sex, hypertension, dyslipidemia, hyperglycemia, obesity, and smoking are major determinants of *macrovascular* diseases (e.g. stroke, myocardial infarction and peripheral artery disease) [14], and have been shown to act through inducing large artery endothelial dysfunction [15, 16]. However, this does not necessarily imply that microvascular (endothelial) function is affected similarly, as microvascular function is the result of the complex interrelationships among structure and function of vessel wall components (matrix, smooth muscle cells, and endothelium), which are also closely related to metabolic and neurogenic influences [17–21]. In addition, endothelial cells are known to be heterogeneous, depending on their localization [22]. Nevertheless, many of the risk factors for macrovascular diseases are also associated with microvascular diseases [23–26]. Thus, we hypothesized that these risk factors may also be determinants of microvascular (endothelial) function.

Therefore, this study aimed to determine, in a population-based setting, whether cardiovascular risk factors were associated with, and thus potential determinants of, retinal and skin microvascular (endothelial) function. We chose retina and skin because these are unique sites enabling direct and reproducible [27, 28] assessment of microvascular function, as measured by flicker light-induced retinal arteriolar dilation and heat-induced skin hyperemia.

## Methods

### Study population and design

We used data from The Maastricht Study, an observational prospective population-based cohort study. The rationale and methodology have been described previously [29]. In brief, the study focuses on the etiology, pathophysiology, complications and comorbidities of type 2 diabetes and is characterized by an extensive phenotyping approach. Eligible for participation were all individuals aged between 40 and 75 years and living in the southern part of the Netherlands. Participants were recruited through mass media campaigns and from the municipal registries and the regional Diabetes Patient Registry via mailings. Recruitment was stratified according to known type 2 diabetes status, with an oversampling of individuals with type 2 diabetes, for reasons of efficiency. The present report includes cross-sectional data from the first 3451 participants, who completed the baseline survey between November 2010 and September 2013. The examinations of each participant were performed within a time window of three months. The study has been approved by the institutional medical ethical committee (NL31329.068.10) and the Minister of Health, Welfare and Sports of the Netherlands (Permit 131088-105234-PG). All participants gave written informed consent.

### Assessment of microvascular function

All participants were asked to refrain from smoking and drinking caffeine-containing beverages three hours before the measurement. A light meal (breakfast and (or) lunch), low in fat content, was allowed if taken at least 90 minutes prior to the start of the measurements.

Retinal arteriolar vasodilation to flicker light exposure was measured by the Dynamic Vessel Analyzer (Imedos, Jena, Germany). Briefly, a baseline recording of 50 seconds was followed by 40-second flicker light exposure followed by a 60-second recovery period. Baseline diameter was calculated as the average diameter size of the 20–50 seconds recording and was expressed in measurement units (MU). Percentage dilation over baseline was based on the average dilation achieved at time points 10 and 40 seconds during the flicker stimulation period.

Heat-induced skin hyperemia was measured by laser-Doppler flowmetry (Perimed, Järfälla, Sweden). Briefly, skin blood flow, expressed in arbitrary perfusion units (PU), was recorded unheated for 2 minutes to serve as a baseline. After 2 minutes, the temperature of the laser-Doppler probe was rapidly and locally increased to 44°C, and was kept constant until the end of the registration. The heat-induced skin hyperemic response was expressed as the percentage increase in average PU during the 23 minutes heating phase over the 2 minutes average baseline PU. Both measurements have extensively been described previously [30]; more details are provided in the S1 Appendix.

### Definition of cardiovascular risk factors

Cardiovascular risk factors considered were age, sex, waist circumference, fasting plasma glucose (FPG), total-to-high-density lipoprotein (HDL) cholesterol ratio, 24-h systolic blood pressure (SBP), and smoking status. We also considered lipid-modifying and antihypertensive medication.

### Measurement of cardiovascular risk factors

We determined body mass index, waist circumference, glucose levels, glycated hemoglobin A1c (HbA1c), 24-h ambulatory systolic and diastolic blood pressure (DBP), total and HDL cholesterol, and triglycerides as described previously [29] and detailed in the S2 Appendix. Smoking status (never, former, current) and pack-years of smoking were assessed by web-based questionnaire [29]. Glucose metabolism status was defined according to the World Health Organization 2006 criteria, based on a standardized 2-h 75 gram oral glucose tolerance test (S2 Appendix).

### Measurement of covariates

The use of lipid-modifying and antihypertensive medication was assessed during a medication interview where generic name, dose, and frequency were registered [29]. The assessment of a history of cardiovascular disease (CVD), 24-h urinary albumin excretion, estimated glomerular filtration rate (eGFR), and the presence of retinopathy have been described previously [29] (cf. the S3 Appendix).

### Statistical analysis

All analyses were performed with Statistical Package for Social Sciences version 23.0 (IBM SPSS, Armonk, USA). Variables with a skewed distribution (diabetes duration and pack-years of smoking) were log10 transformed. A Pearson correlation coefficient was used to assess the correlation between flicker light-induced retinal arteriolar %-dilation and heat-induced skin %-hyperemia. Standardized multiple linear regression analyses were used to evaluate the association of cardiovascular risk factors (age, sex, waist circumference, FPG, total-to-HDL cholesterol ratio, 24-h SBP, and smoking status) with both retinal and skin microvascular function. To compare regression coefficients between cardiovascular risk factors, continuous measures of these risk factors were standardized into z-scores before analyses. Associations of cardiovascular risk factors were adjusted for each other and for additional covariates (the use of antihypertensive and lipid-modifying medication, a history of CVD, 24-h urinary albumin excretion, eGFR, and the presence of retinopathy). In additional analyses, FPG was substituted by HbA1c, 2-h postload or by type 2 diabetes (T2DM) (yes/no). Waist circumference was substituted by body mass index, and 24-h SBP by 24-h DBP, 24-h mean arterial pressure or 24-h pulse pressure.

Data were expressed as standardized regression coefficients and their 95% confidence interval (95%CI). A P-value <0.05 was considered statistically significant. The study, by design, oversampled individuals with T2DM; we therefore investigated potential interactions between cardiovascular risk factors and T2DM by adding interaction terms (the product of a cardiovascular risk factor and T2DM) to the regression models. Similarly, interactions between the cardiovascular risk factors and sex were investigated (the product of a cardiovascular risk factor and sex). A P_interaction_<0.10 was considered statistically significant. A P_interaction_≥0.10 indicates that the association between a cardiovascular risk factor and retinal or skin microvascular function does not statistically significantly differ between individuals without and with T2DM, or between women and men, respectively [31]. A non-significant P_interaction_ between a cardiovascular risk factor and T2DM therefore indicates that the association between that cardiovascular risk factor and retinal or skin microvascular function was not driven by the oversampling of individuals with T2DM. This implies that any associations observed in this T2DM-enriched population can be considered valid for a non-oversampled population, i.e. the general population [31]. Collinearity diagnostics (i.e. tolerance <0.10 and/or variance inflation factor >10) were used to detect multicollinearity between the cardiovascular risk factors and covariates.

## Results

### Study population

**Fig 1:**
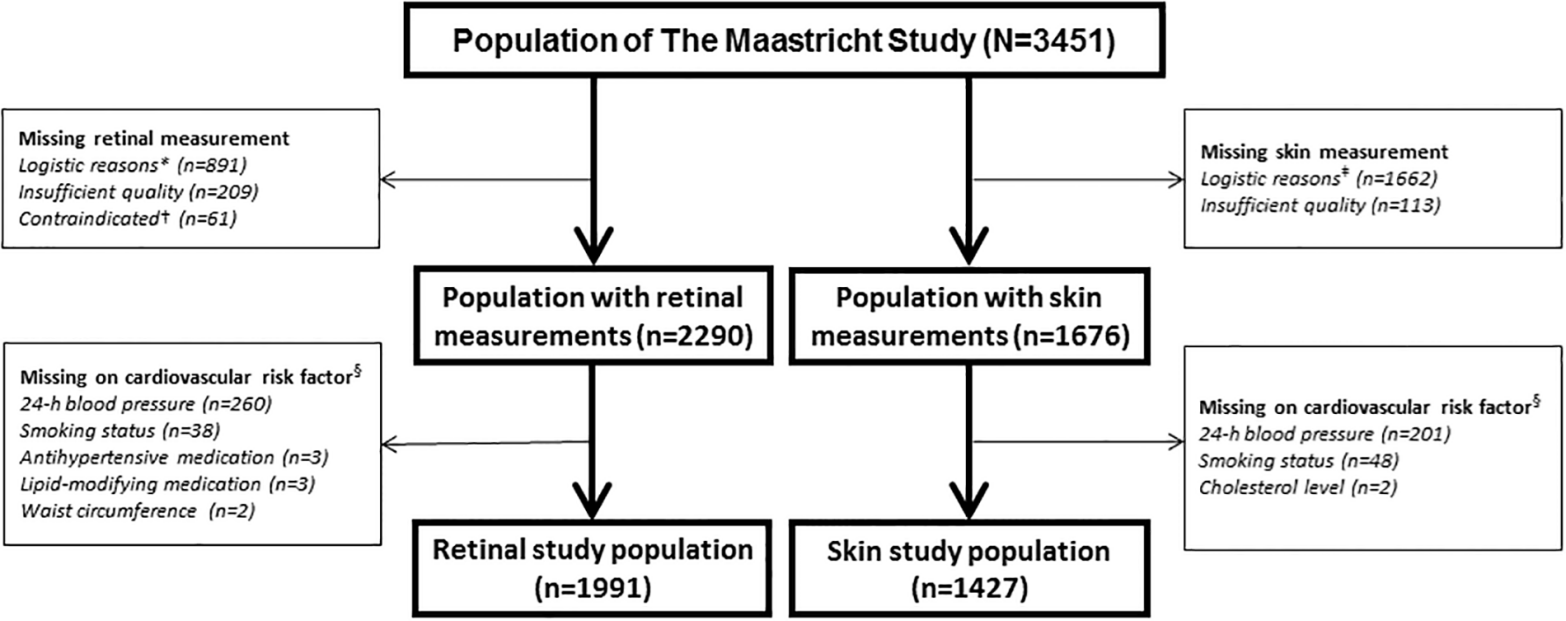
Retinal and skin study population selection. * = Logistical reasons: no Dynamic Vessel Analyzer equipment available (n = 536), no trained researcher available (n = 232), no eye drops given for traffic safety reasons (n = 123); † = Contraindicated: history of epilepsy (n = 14), allergy to eye drops (n = 33), glaucoma or lens implants (n = 14); ‡ = Logistical reasons: no laser-Doppler equipment available (n = 354), no trained researcher available (n = 271), technical failure (n = 1037). § = Missing values on cardiovascular risk factors were not mutually exclusive.

From the initial 3451 participants, retinal arteriolar reactivity data was available in 2290. The reasons for missing data were logistical (n = 891), insufficient measurement quality (n = 209) or contraindications (n = 61). Data on cardiovascular risk factors were missing in 299 participants, particularly on 24-h blood pressure (n = 260), mainly due to device availability. The population in which retinal arteriolar reactivity data were available thus consisted of 1991 participants. Heat-induced skin hyperemia data were available in 1676 of the 3451 participants. The reasons for missing data were logistical (n = 1662) or insufficient measurement quality (n = 113). Cardiovascular risk factors were missing in 249 participants, mainly due to missing 24-h blood pressure values (n = 201). The population in which heat-induced skin hyperemia data were available thus consisted of 1427 participants (Fig 1 shows the flow chart).

Table 1 shows general characteristics of the retinal arteriolar reactivity study population stratified into tertiles of retinal arteriolar %-dilation. This study population had a mean ± standard deviation (SD) age of 59.7±8.2 years, 48.8% were women, 12.1% were current smokers, and 27.4% had T2DM. In addition, when compared to individuals in the lowest tertile of retinal arteriolar %-dilation, those in the middle and highest tertiles were on average younger and had lower fasting and 2-h postload glucose levels (Table 1). The skin study population overlapped for 73% with the retinal study population, and was comparable with regard to age, sex, and cardio-metabolic risk profile (Table 1). The Pearson correlation coefficients between retinal arteriolar %-dilation and skin %-hyperemia was Pearson’s r = 0.05, P = 0.095. Individuals with missing data on retinal or skin reactivity measurements or measurements of cardiovascular risk factors were generally comparable to individuals included in the study populations with regard to age, sex, and cardiometabolic risk profile (S1 and S2 Tables).

**Table 1:** General characteristics of the retinal and skin study populations according to tertiles of retinal arteriolar %-dilation and skin %-hyperemia.

	Retinal study population				Skin study population			
Characteristic	Tertile 1 of retinal arteriolar dilation (lowest) n = 662	Tertile 2 of retinal arteriolar dilation n = 665	Tertile 3 of retinal arteriolar dilation (highest) n = 664	P for trend	Tertile 1 of skin hyperemia (lowest) n = 475	Tertile 2 of skin hyperemia n = 476	Tertile 3 of skin hyperemia (highest) n = 476	P for trend
Range of retinal arteriolar %-dilation	-4.8 to 1.3	1.3 to 4.0	4.0 to 15.2		-	-	-	-
Range of skin %-hyperemia	-	-	-	-	20.8 to 725.8	725.9 to 1286.6	1286.6 to 6763.6	
Age (years)	61.5±7.6	59.4±8.3	58.3±8.4	<0.001	61.3±7.7	60.8±7.9	58.5±8.4	<0.001
Women	289 (43.7)	345 (51.9)	337 (50.8)	0.005	164 (34.5)	212 (44.5)	298 (62.6)	<0.001
- Postmenopausal	234 (84.5)	256 (79.0)	236 (72.0)	<0.001	126 (84.0)	154 (79.4)	198 (72.5)	<0.001
- Hormone replacement therapy	6 (0.9)	6 (0.9)	5 (0.8)	0.942	2 (0.4)	4 (0.8)	9 (1.9)	0.073
Glucose metabolism status				<0.001				<0.001
- Normal glucose metabolism	305 (46.1)	388 (58.3)	438 (66.0)		216 (45.5)	249 (52.3)	298 (62.6)	
- Prediabetes	102 (15.4)	92 (13.8)	98 (14.8)		74 (15.6)	70 (14.7)	78 (16.4)	
- Type 2 diabetes	242 (36.6)	178 (26.8)	125 (18.8)		178 (37.5)	146 (30.7)	96 (20.2)	
- Other types of diabetes	13 (2.0)	7 (1.1)	3 (0.5)		7 (1.5)	11 (2.3)	4 (0.8)	
Type 2 diabetes duration (years)	6.0 [3.0–13.0]	5.0 [3.0–10.0]	6.0 [3.0–13.0]	0.223	5.5 [3.0–10.5]	7.0 [3.0–13.0]	6.0 [3.0–10.0]	0.518
Body mass index (kg/m^2^)	27.4±4.4	26.7±4.7	26.5±4.2	0.001	26.9±4.1	27.2±4.4	26.7±4.6	0.220
Weight (kg)	80.1±15.5	78.4±16.2	78.2±15.4	0.047	79.7±14.7	80.3±15.4	77.2±14.7	0.003
Height (cm)	170.8±8.7	171.0±9.0	171.4±8.8	0.399	171.9±8.5	171.5±8.7	169.9±8.7	0.001
Waist circumference (cm)								
- Men	102.6±12.3	100.6±12.2	99.9±10.8	0.007	101.4±11.9	101.7±11.7	99.2±11.1	0.063
- Women	89.9±12.2	89.2±13.1	88.0±12.2	0.162	88.8±12.2	90.6±12.6	90.4±13.3	0.329
History of cardiovascular disease	132 (20.2)	101 (15.4)	73 (11.1)	<0.001	91 (19.4)	86 (18.4)	72 (15.3)	0.227
Office SBP (mmHg)	136.7±18.0	134.0±18.4	134.3±17.5	0.013	136.6±17.4	136.0±18.6	134.6±18.4	0.210
Office DBP (mmHg)	75.7±9.8	76.2±10.3	76.9±9.6	0.070	76.3±9.4	76.4±9.8	76.7±9.8	0.770
Ambulatory 24-h SBP (mmHg)	119.6±11.5	119.0±12.4	118.9±10.9	0.477	121.8±11.9	120.0±11.9	117.5±10.8	<0.001
Ambulatory 24-h DBP (mmHg)	72.8±7.1	73.6±7.5	74.1±6.8	0.002	74.0±7.1	73.5±7.0	73.4±6.5	0.375
Ambulatory 24-h PP (mmHg)	46.8±8.9	45.4±8.8	44.7±7.7	<0.001	47.8±9.1	46.6±9.0	44.1±8.1	<0.001
Ambulatory 24-h MAP (mmHg)	88.4±7.8	88.8±8.5	89.0±7.5	0.305	89.9±7.9	89.0±7.9	88.1±7.2	0.002
Smoking								
- Never / former / current	198/386/78	248/328/89	253/337/74	0.005	152/244/79	137/286/53	180/251/45	<0.001
- % (never / former / current)	29.9/58.3/11.8	37.3/49.3/13.4	38.1/50.8/11.1	0.005	32.0/51.4/16.6	28.8/60.1/11.1	37.8/52.7/9.5	<0.001
Pack-years of smoking	4.6 [0.0–20.0]	1.0 [0.0–17.2]	0.7 [0.0–13.0]	0.001	4.6 [0.0–22.2]	6.0 [0.0–22.5]	1.3 [0.0–14.0]	<0.001
Fasting glucose (mmol/l)	6.4±2.0	6.0±1.6	5.8±1.3	<0.001	6.4±1.9	6.1±1.6	5.8±1.3	<0.001
2-h postload glucose (mmol/l)	8.6±4.6	7.9±4.2	7.3±3.8	<0.001	8.8±4.7	8.2±4.2	7.3±4.0	<0.001
HbA1c (%)	6.1±1.1	5.8±0.9	5.7±0.7	<0.001	6.1±1.1	6.0±0.9	5.8±0.8	<0.001
HbA1c (mmol/mol)	43.3±11.8	40.3±9.5	38.8±7.9	<0.001	43.7±11.7	41.8±9.5	39.9±8.7	<0.001
Total-to-HDL cholesterol ratio	3.5±1.1	3.6±1.2	3.6±1.2	0.168	3.8±1.1	3.7±1.2	3.6±1.1	0.181
Total cholesterol (mmol/l)	5.0±1.2	5.3±1.2	5.4±1.1	<0.001	5.1±1.1	5.2±1.2	5.4±1.2	<0.001
HDL cholesterol (mmol/l)	1.5±0.5	1.6±0.5	1.6±0.5	0.019	1.4±0.5	1.5±0.5	1.6±0.5	<0.001
LDL cholesterol (mmol/l)	2.9±1.0	3.1±1.0	3.2±1.0	<0.001	2.9±1.0	3.1±1.1	3.3±1.1	<0.001
Triglycerides (mmol/l)	1.5±0.8	1.5±0.9	1.3±0.7	0.001	1.5±0.8	1.5±1.0	1.3±0.8	<0.001
Antihypertensive medication use	305 (46.1)	257 (38.6)	198 (29.8)	<0.001	219 (46.1)	211 (44.3)	163 (34.2)	<0.001
Lipid-modifying medication use	309 (46.7)	221 (33.2)	176 (26.5)	<0.001	209 (44.0)	202 (42.4)	146 (30.7)	<0.001
Diabetes medication use								
- Any type	205 (31.0)	143 (21.5)	98 (14.8)	<0.001	147 (30.9)	127 (26.7)	77 (16.2)	<0.001
- Insulin	71 (10.7)	37 (5.6)	24 (3.6)	<0.001	42 (8.8)	41 (8.6)	22 (4.6)	0.020
- Oral glucose-lowering medication	180 (27.2)	131 (19.7)	87 (13.1)	<0.001	132 (27.8)	112 (23.5)	67 (14.1)	<0.001
eGFR (ml/min/1.73m^2^)	86.7±15.8	88.5±14.4	89.5±13.5	0.001	87.4±14.7	88.0±14.8	89.7±13.8	0.044
eGFR<60 ml/min/1.73m^2^	45 (6.8)	25 (3.8)	15 (2.3)	<0.001	15 (3.2)	19 (4.0)	17 (3.6)	0.780
(Micro)albuminuria*	78 (11.8)	42 (6.4)	43 (6.5)	<0.001	50 (10.6)	42 (8.9)	25 (5.3)	0.011
Retinopathy	20 (3.1)	10 (1.6)	2 (0.3)	<0.001	7 (1.6)	11 (2.6)	3 (0.7)	0.099
Baseline arteriolar diameter (MU)	119.4±15.0	115.3±15.5	111.6±15.2	<0.001	-	-	-	-
Arteriolar average dilation (%)								
- Mean±SD	0.2±0.9	2.6±0.8	6.3±1.8	<0.001	-	-	-	-
- Median (interquartile range)	0.4 [-0.2–0.8]	2.6 [1.9–3.3]	5.9 [4.9–7.2]	<0.001	-	-	-	-
Baseline skin blood flow (PU)	-	-	-	-	15.5±8.5	9.9±4.0	7.7±2.6	<0.001
Skin hyperemic response (%)								
- Mean±SD	-	-	-	-	418.2±191.3	992.2±158.9	1976.8±715.6	<0.001
- Median (interquartile range)	-	-	-	-	423.5 [256.1–586.2]	1002.0 [852.4–1119.0]	1757.7 [1508.7–2196.2]	<0.001

Data are reported as mean±SD, median [interquartile range], or number (percentages %) as appropriate. SBP, systolic blood pressure; DBP, diastolic blood pressure; MAP, mean arterial pressure; PP, pulse pressure; HbA1c, glycated hemoglobin A1c; HDL, high-density lipoprotein; LDL, low-density lipoprotein; eGFR, estimated glomerular filtration rate; MU, measurement units; PU, perfusion units; SD, standard deviation. P for trend as determined with use of one-way ANOVA for continuous variables and χ^2^-test for categorical variables.

* = (Micro)albuminuria was defined as a urinary albumin excretion of >30 mg per 24 hours.

### Age, sex and retinal arteriolar dilation and skin hyperemia

**Fig 2:**
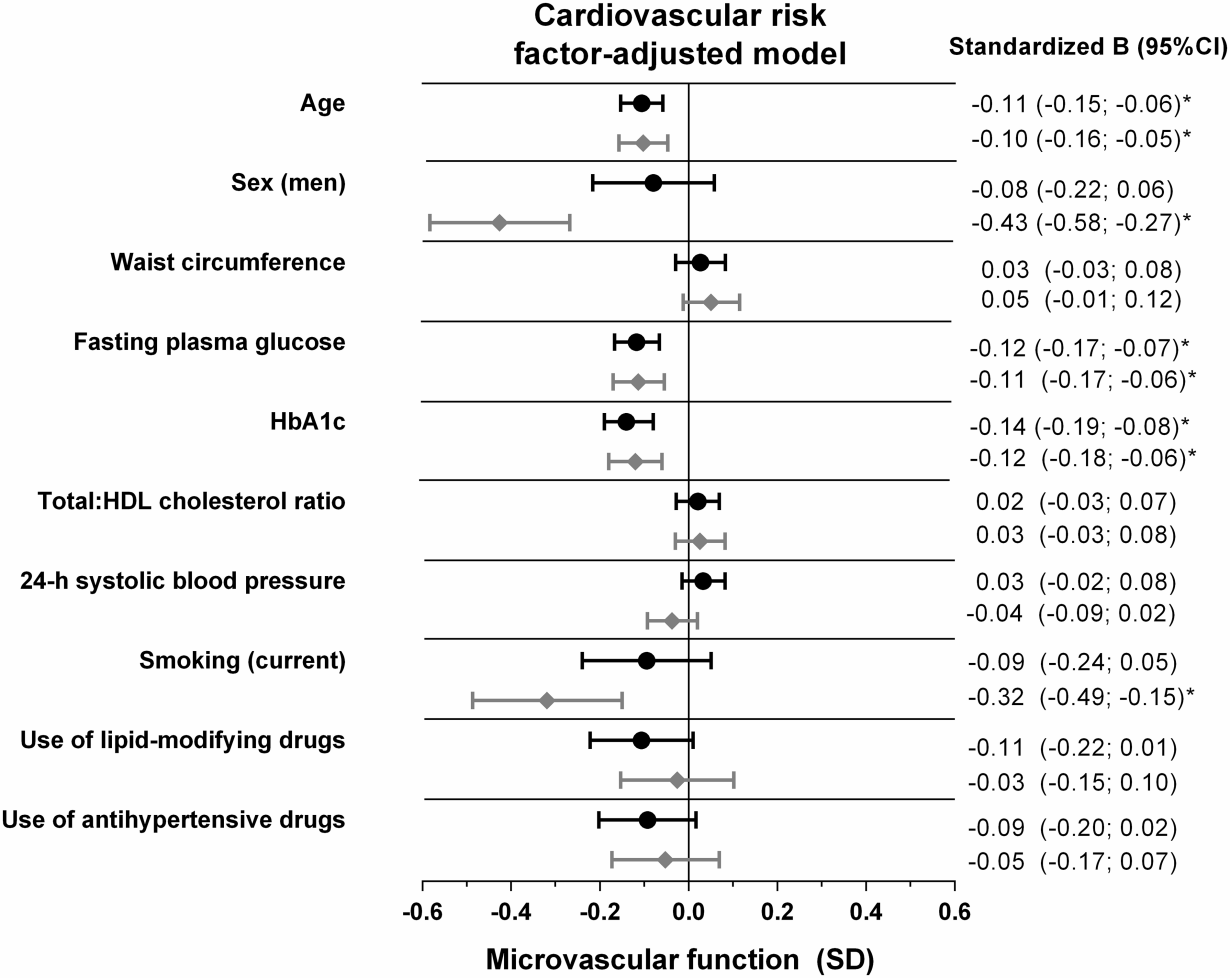
Associations of cardiovascular risk factors with retinal arteriolar %-dilation and skin %-hyperemia. Point estimates (standardized beta) and 95%CIs represent the difference (in SD) in retinal arteriolar %-dilation (black dots) and skin %-hyperemia (grey diamonds) per SD increase in the cardiovascular risk factor, men versus women, current smoker versus never smoker, or the use of antihypertensive or lipid-modifying medication versus no use. All associations were adjusted for the other risk factors, except for HbA1c, with multivariate regression. Associations of HbA1c were based on a fully adjusted regression model in which fasting plasma glucose was replaced by HbA1c. Associations of sex were additionally adjusted for height. *P<0.05, SD, standard deviation; CI, confidence interval; HDL, high-density lipoprotein; HbA1c, glycated hemoglobin A1c.

**Fig 3:**
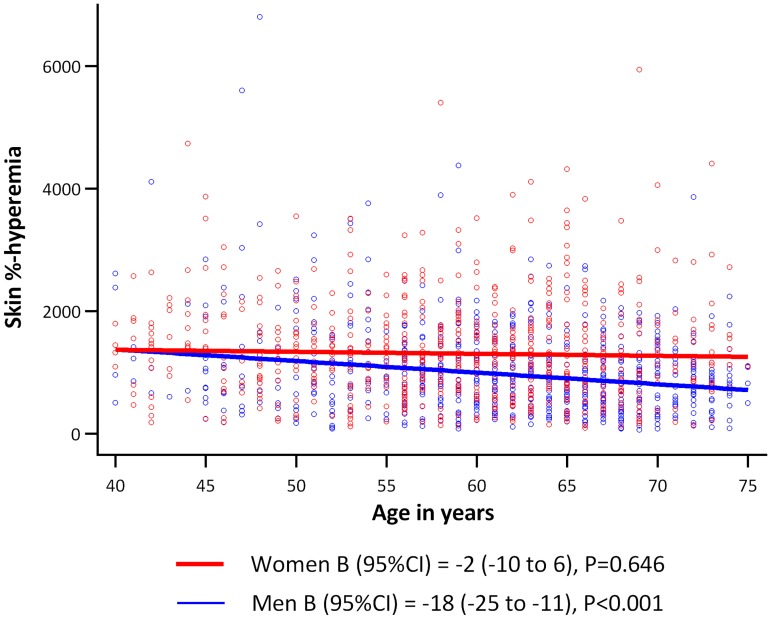
Association between age and skin %-hyperemia according to sex. Regression coefficients (B) indicate the adjusted mean difference and 95% confidence interval (95%CI) in skin %-hyperemia per 1 year increase in age for men (blue line) and women (red line) (P_interaction_ = 0.002).

Age was inversely associated with retinal arteriolar %-dilation and skin %-hyperemia; per SD higher age (8.2 years), retinal arteriolar %-dilation was -0.11SD (95%CI: -0.15; -0.06, P<0.001) lower, and skin %-hyperemia was -0.10SD (-0.16; -0.05, P<0.001) lower. Sex was not associated with retinal arteriolar %-dilation, whereas men had a -0.43SD (-0.58; -0.27, P<0.001) lower skin %-hyperemic response as compared to women (Fig 2, and S3 and S4 Tables). Moreover, we observed a sex-by-age interaction in which the inverse association between age and skin %-hyperemia was stronger in men (-0.21SD (-0.29; -0.13), P<0.001) than in women (-0.02SD (-0.10; 0.06), P = 0.646) (P_interaction_ = 0.002, Fig 3). Additional adjustment for postmenopausal status and/or hormone replacement therapy in women did not materially change these associations (data not shown).

### Glycemia and retinal arteriolar dilation and skin hyperemia

FPG (as a measure of short-term hyperglycemia) was inversely associated with retinal arteriolar %-dilation and skin %-hyperemia; per SD higher FPG (1.7 mmol/l), retinal arteriolar %-dilation was -0.12SD (95%CI: -0.17; -0.07, P<0.001) lower, and skin %-hyperemia was -0.11SD (-0.17; -0.06, P<0.001) lower (Fig 2, and S3 and S4 Tables). In addition, HbA1c (as a measure of long-term hyperglycemia, substituted for FPG) was inversely associated with retinal arteriolar %-dilation and skin %-hyperemia; per SD higher HbA1c (0.9 mmol/l), retinal arteriolar %-dilation was -0.14SD (95%CI: -0.19; -0.08, P<0.001) lower, and skin %-hyperemia was -0.12SD (-0.18; -0.06, P<0.001) lower (S5 and S6 Tables).

### Smoking and retinal arteriolar dilation and skin hyperemia

Current smoking (versus never smoking) was not associated with retinal arteriolar %-dilation, whereas it was associated with -0.32SD (-0.49; -0.15, P<0.001) lower skin %-hyperemia (Fig 2, and S3 and S4 Tables). Former smoking (versus never smoking) was not associated with retinal arteriolar %-dilation and skin %-hyperemia.

### Blood pressure and retinal arteriolar dilation and skin hyperemia

24-h SBP was not associated with retinal arteriolar %-dilation or skin %-hyperemia (Fig 2, and S3 and S4 Tables). However, 24-h DBP (substituted for 24-h SBP) was associated with retinal arteriolar %-dilation; per SD higher 24-h DBP (7.2 mmHg), retinal arteriolar %-dilation was 0.05SD (0.00; 0.10, P = 0.040) greater. 24-h DBP was not associated with skin %-hyperemia. In addition, 24-h pulse pressure (substituted for 24-h SBP and additionally corrected for 24-h mean arterial pressure) was not associated with retinal arteriolar %-dilation, but was inversely associated with skin %-hyperemia. Per SD higher 24-h pulse pressure (8.9 mmHg), skin %-hyperemia was -0.08SD (-0.14; -0.01, P = 0.017) lower. In addition, use of antihypertensive medication was associated with numerically lower retinal arteriolar %-dilation (-0.09SD (-0.20; 0.02), P = 0.099) and skin %-hyperemia (-0.05SD (-0.17; 0.07), P = 0.396).

### Waist, total-to-HDL cholesterol, and lipid-modifying medication use and retinal arteriolar dilation and skin hyperemia

No significant associations were observed between waist circumference and total-to-HDL cholesterol ratio with retinal arteriolar %-dilation or skin %-hyperemia. The use of lipid-modifying medication was associated with numerically lower retinal arteriolar %-dilation (-0.11SD (-0.22; 0.01), P = 0.072)(Fig 2 and S3 Table).

### Additional analyses

Qualitatively similar associations of cardiovascular risk factors with retinal arteriolar %-dilation and skin %-hyperemia were observed in a range of additional analyses. First, when flicker light-induced increase (in MU) in retinal arteriolar diameter from baseline or heat-induced increase in skin blood flow (in PU) from skin baseline were used rather than their percentages (data not shown). Second, when waist circumference was replaced by body mass index. Third, when FPG was substituted by 2-h postload glucose or by type 2 diabetes (yes/no). Fourth, when smoking status (never/former/current) was replaced by pack-years of smoking (for retinal and skin analyses, data on pack-years of smoking were available in 1672 and 1231 individuals, respectively); pack-years of smoking was inversely associated with skin %-hyperemia -0.07 SD (-0.12; -0.01, P = 0.020). No association was found between pack-years of smoking and retinal arteriolar %-dilation (data not shown). Fifth, after additional adjustment for glucose-lowering medication (although this may be an overadjustment as the use of glucose-lowering medication was part of the definition of type 2 diabetes (78% of individuals with type 2 diabetes used glucose-lowering medication)). Sixth, after additional adjustment for a history of CVD, eGFR, urinary albumin excretion, and the presence of retinopathy (data not shown, for retinal and skin analyses, data on these additional covariates were available in 1884 and 1254 individuals respectively). Seventh, when antihypertensive medication was further specified into renin-angiotensin-aldosterone system (RAAS)-inhibiting (with or without other types of antihypertensives) and non-RAAS-inhibiting antihypertensives only (data not shown). RAAS-inhibiting antihypertensives included angiotensin-converting-enzyme, angiotensin receptor blockers and renin blockers. Eight, when individuals with other types of diabetes than type 2 diabetes were excluded (for retinal and skin analyses, 23 and 22 individuals with other types of diabetes were excluded, respectively). Next, associations of cardiovascular risk factors with retinal arteriolar %-dilation and skin %-hyperemia did not differ between individuals with and without type 2 diabetes, or between women and men (all P_interactions_>0.10), except as noted above, for the significant interaction, with sex, of the association between age and heat-induced skin hyperemia (Fig 3). Last, collinearity diagnostics revealed no multicollinearity in any of the analyses (i.e. all tolerance values ≥0.10 and variance inflation factors ≤10).

## Discussion

To our knowledge this is the first population-based study which demonstrated that associations between cardiovascular risk factors and retinal and skin microvascular (endothelial) function show a pattern that is in part similar to the associations between cardiovascular risk factors and *macrovascular* function [14, 16]. Thus, older age and measures of hyperglycemia were associated with an impaired retinal and skin microvascular vasodilation response, independent of other cardiovascular risk factors. In addition, male sex and cigarette smoking were associated with impaired heat-induced skin hyperemia. In contrast to associations of obesity, blood pressure and lipid profile with *macrovascular* endothelial function [16], we could not confirm waist circumference, body mass index, 24-h SBP, and total-to-HDL cholesterol ratio as determinants of *microvascular* function. However, (inverse) associations of antihypertensive and lipid-modifying medication use with retinal and skin microvascular function could not be excluded (Fig 2). Such associations may imply that previous exposure to elevated blood pressure and dyslipidemia may affect microvascular function more than actual blood pressure and lipid profile. This would also explain why no associations of 24-h SBP and total-to-HDL cholesterol ratio with microvascular function were found.

An important mechanism by which cardiovascular risk factors affect large artery endothelial vasodilation is by impairing nitric oxide bioavailability [32], possibly in conjunction with adverse effects on other endothelium-dependent vasodilators (e.g. endothelium-derived hyperpolarizing factors) [33]. Similar mechanisms may apply to microvascular function, as microvascular vasodilation also relies on availability of endothelium-dependent vasodilators [19, 34]. Therefore, risk factor-associated impairments in flicker light-induced retinal arteriolar dilation and heat-induced skin hyperemia are both likely to be a reflection of microvascular endothelial dysfunction [19, 34], presumably in conjunction with vascular smooth muscle cell dysfunction [20, 21], and/or neuronal dysfunction [18, 35]. The correlation between these measures was weak and not statistically significant, which was likely to be explained by 1) different vessel types (relatively large arterioles in the retina vs small arterioles, capillaries, and venules in skin); 2) different outcomes (a direct stimulus-induced increase in diameter in the retina vs, an indirect estimate of vasodilation by measuring stimulus-induced increase in perfusion in skin); and 3) different stimuli used to elicit the responses (flicker light vs heat) [18, 19, 34, 35]. In addition, both measures of microvascular function have good reproducibility with intra-individual coefficients of variation of 0.91% for flicker light-induced retinal arteriolar dilation [28] and 8.7% for heat-induced skin hyperemia [27].

Associations between risk factors and microvascular responses were generally similar regardless of whether the microvascular response was obtained in retina or skin, with two exceptions. First, no difference was found between men and women in retinal arteriolar dilation, whereas heat-induced skin hyperemia was less in men than in women. This is in line with an earlier study which showed that sex differences in retinal arteriolar dilation (i.e. greater in men) were present in young individuals, but diminished after the age of 30 years [36]. This may explain why, in our study with an age span of 40–75 years, no difference was found. Greater retinal arteriolar dilation in younger men, as compared to age-matched women [36], contrasts with the beneficial effect of female sex hormones on ocular and skin blood flow [13, 37]. However, the retinal arteriolar dilation response depends on neurovascular coupling [38], and sex differences thus may also be influenced by the effects of sex hormones on neurons and astrocytes in the neurovascular coupling unit [39]. Second, retinal arteriolar dilation decreased with age in both men and women, whereas heat-induced skin hyperemia decreased with age only in men (Fig 3), which is consistent with an earlier report on macrovascular endothelial function [40]. Thus, heat-induced skin hyperemia, as compared to retinal arteriolar dilation, may be protected more by the higher levels of estrogens in women than in men [36, 41, 42].

Age was inversely associated with retinal arteriolar and skin microvascular dysfunction, which is in line with earlier smaller studies [8, 13], and is likely caused by reduced nitric oxide availability [43]. In turn, hyperglycemia may impair microvascular function through intraendothelial accumulation of glucose, increased oxidative stress and formation of advanced glycation end products [44]. In addition, microvascular dysfunction can cause hyperglycemia by impairing the timely access of glucose and insulin to their target tissue [45] and by impairing insulin secretion [46].

We hypothesized, but did not find, higher blood pressures to be *consistently* associated with impaired microvascular function [8, 11, 13], especially in the retina, which is known to be sensitive to greater flow pulsatility associated with higher blood pressure and arterial stiffening [47]. A potential explanation is that, in this relatively healthy and well-treated population, the blood pressure range was insufficiently broad for such associations to appear, except that 24-h pulse pressure was inversely associated with heat-induced skin hyperemia. Interestingly, use of antihypertensive medication was associated with numerically lower microvascular function, suggesting that prior exposure to high blood pressure may be important.

Current, but not former smoking was associated with impaired heat-induced skin hyperemia, which suggests that effects of smoking may be reversible. These findings are in line with an earlier report on the detrimental effect of smoking on acetylcholine-induced skin hyperemia [10]. Mechanistically, smoking may induce microvascular dysfunction via increased formation of reactive oxygen species and/or inhibition of nitric oxide synthase activity [48]. As demonstrated previously [49], we also did not observe a clear association of smoking with impaired retinal arteriolar dilation. Possibly, smoking affects smaller arterioles and capillaries (such as those involved in heat-induced skin hyperemia) more than the relatively large retinal arterioles we assessed [49].

We hypothesized [7, 45], but did not find, higher waist circumference and body mass index to be associated with impaired microvascular function as assessed here. Importantly, these findings do not imply that other functions of the microcirculation are normal in overweight or obese individuals. Indeed, earlier reports have shown waist and body mass index to be inversely associated with microvascular vasomotion [50], post-occlusive reactive hyperemia [51], and insulin-mediated vasodilation [45], mediated presumably by changes in visceral and perivascular [52] adipose tissue-derived factors, such as increased tumor necrosis factor-α and free fatty acids, and decreased adiponectin [45].

We expected [9, 53], but did not find, inverse associations between total-to-HDL cholesterol ratio and retinal and skin microvascular function as assessed here. Mechanistically, dyslipidemia may impair microvascular vasodilation via oxidative modifications of low-density lipoprotein cholesterol, which may cause reduced nitric oxide availability, possibly in conjunction with increased expression of endothelin-1 [53]. Interestingly, use of lipid-modifying medication was associated with numerically lower retinal arteriolar dilation, suggesting that prior exposure to dyslipidemia may be important.

Strengths of our study include its size and population-based design; the extensive assessment of potential determinants, including 24-h ambulatory blood pressure; the use of two independent methods to directly assess microvascular function in different microvascular beds; and the broad array of additional analyses, which all gave consistent results.

Our study also had limitations. First, the data were cross-sectional. Reverse causality obviously is not an issue for associations with age and sex, but may be especially relevant for hyperglycemia and blood pressure [54]. Therefore, longitudinal studies are needed. Second, we mainly focused on major cardiovascular risk factors as potential determinants; however, we do not claim to have identified all determinants, as there may be others not included in these analyses (e.g. dietary habits and physical activity). Last, the associations observed do not imply that other estimates of microvascular function, or retinal and skin microvascular responses elicited via different stimuli, such as exercise, insulin or ischemia, relate similarly to cardiovascular risk factors, as compared to flicker light-induced retinal arteriolar dilation and heat-induced skin hyperemia.

This population-based study demonstrated that associations between cardiovascular risk factors and retinal and skin *microvascular* (endothelial) function show a pattern that is in part similar to the associations between cardiovascular risk factors and *macrovascular* endothelial function. Thus, age and measures of hyperglycemia were inversely associated with retinal and skin microvascular vasodilation. In addition, male sex and cigarette smoking were associated with impaired heat-induced skin hyperemia. All associations were independent of the other cardiovascular risk factors. We could not confirm waist circumference, body mass index, 24-h SBP, and total-to-HDL cholesterol ratio as determinants of these microvascular functions. Possibly, prior exposure to high blood pressure and/or dyslipidemia is important as use of antihypertensive and/or lipid-modifying medication was associated with numerically lower microvascular function. We conclude that impairment of microvascular function may constitute a pathway through which an adverse cardiovascular risk factor pattern may increase risk of diseases, such as heart failure, stroke, and cognitive decline, that in part have a microvascular origin.

## Supporting information

S1 Appendix Assessment of retinal and skin microvascular function. (DOCX)

S2 Appendix Details on the assessment of cardiovascular risk factors. (DOCX)

S3 Appendix Details on the assessment of covariates. (DOCX)

S1 Table General characteristics for the retinal reactivity study population and individuals excluded from the analyses due to missing values. Data are reported as mean ± SD, median [interquartile range], or number (percentages %) as appropriate. P-value indicates comparison between study population and individuals excluded due to missing values. SD, standard deviation; SBP, systolic blood pressure; DBP, diastolic blood pressure; PP, pulse pressure; MAP, mean arterial pressure; HbA1c, glycated hemoglobin A1c; HDL, high-density lipoprotein; LDL, low-density lipoprotein; eGFR, estimated glomerular filtration rate; MU, measurement units. * = Total number of missing values for a specific variable in the retinal reactivity study population, † = Total number of missing values for a specific variable in the population which was excluded, ‡ = (Micro)albuminuria was defined as a urinary albumin excretion of >30 mg per 24 hours, § = 299 were excluded due to missing on potential cardiovascular risk factors.
(DOCX)

S2 Table General characteristics for the skin hyperemia study population and individuals excluded from the analyses due to missing values. Data are reported as mean ± SD, median [interquartile range], or number (percentages %) as appropriate. P-value indicates comparison between study population and individuals excluded due to missing values. SD, standard deviation; SBP, systolic blood pressure; DBP, diastolic blood pressure; PP, pulse pressure; MAP, mean arterial pressure; HbA1c, glycated hemoglobin A1c; HDL, high-density lipoprotein; LDL, low-density lipoprotein; eGFR, estimated glomerular filtration rate; PU, perfusion units. * = Total number of missing values for a specific variable in the skin hyperemia study population, † = Total number of missing values for a specific variable in the population which was excluded, ‡ = (Micro)albuminuria was defined as a urinary albumin excretion of >30 mg per 24 hours, § = 249 were excluded due to missing on potential cardiovascular risk factors.
(DOCX)

S3 Table Multivariable-adjusted regression analyses of associations between cardiovascular risk factors with retinal arteriolar %-dilation. Point estimates (standardized beta) and 95%CIs represent the difference (in SD) in retinal arteriolar %-dilation per SD increase in the cardiovascular risk factor, men versus women, current smoker versus never smoker, or the use of antihypertensive or lipid-modifying medication versus no use. All associations were adjusted for the other risk factors with multivariate regression. Associations of sex were additionally adjusted for height. Note that as a consequence of standardization of the continuous variables (age, waist circumference, fasting plasma glucose, total-to-HDL cholesterol, 24-h systolic blood pressure) the regression coefficient (B) for continuous variables equals the standardized beta. *P<0.05, SD, standard deviation; CI, confidence interval; HDL, high-density lipoprotein.
(DOCX)

S4 Table Multivariable-adjusted regression analyses of associations between cardiovascular risk factors with skin %-hyperemia. Point estimates (standardized beta) and 95%CIs represent the difference (in SD) in skin %-hyperemia per SD increase in the cardiovascular risk factor, men versus women, current smoker versus never smoker, or the use of antihypertensive or lipid-modifying medication versus no use. All associations were adjusted for the other risk factors with multivariate regression. Associations of sex were additionally adjusted for height. Note that as a consequence of standardization of the continuous variables (age, waist circumference, fasting plasma glucose, total-to-HDL cholesterol, 24-h systolic blood pressure) the regression coefficient (B) for continuous variables equals the standardized beta. *P<0.05, SD, standard deviation; CI, confidence interval; HDL, high-density lipoprotein.
(DOCX)

S5 Table Multivariable-adjusted regression analyses of associations between cardiovascular risk factors with retinal arteriolar %-dilation with fasting plasma glucose substituted by HbA1c. Point estimates (standardized beta) and 95%CIs represent the difference (in SD) in retinal arteriolar %-dilation per SD increase in the cardiovascular risk factor, men versus women, current smoker versus never smoker, or the use of antihypertensive or lipid-modifying medication versus no use. All associations were adjusted for the other risk factors with multivariate regression. Associations of sex were additionally adjusted for height. Note that as a consequence of standardization of the continuous variables (age, waist circumference, HbA1c, total-to-HDL cholesterol, 24-h systolic blood pressure) the regression coefficient (B) for continuous variables equals the standardized beta. *P<0.05, SD, standard deviation; CI, confidence interval; HDL, high-density lipoprotein.
(DOCX)

S6 Table Multivariable-adjusted regression analyses of associations between cardiovascular risk factors with skin %-hyperemia with fasting plasma glucose substituted by HbA1c. Point estimates (standardized beta) and 95%CIs represent the difference (in SD) in skin %-hyperemia per SD increase in the cardiovascular risk factor, men versus women, current smoker versus never smoker, or the use of antihypertensive or lipid-modifying medication versus no use. All associations were adjusted for the other risk factors with multivariate regression. Associations of sex were additionally adjusted for height. Note that as a consequence of standardization of the continuous variables (age, waist circumference, HbA1c, total-to-HDL cholesterol, 24-h systolic blood pressure) the regression coefficient (B) for continuous variables equals the standardized beta. *P<0.05, SD, standard deviation; CI, confidence interval; HDL, high-density lipoprotein.
(DOCX)

## Abbreviations

95%CI: 95% confidence interval
CVD: cardiovascular disease
FPG: fasting plasma glucose
MU: measurement units
PU: perfusion unit
SD: standard deviation

## References

[1] Lee JF, Barrett-O'Keefe Z, Garten RS, Nelson AD, Ryan JJ, Nativi JN, et al Evidence of microvascular dysfunction in heart failure with preserved ejection fraction. *Heart*. 2016;102:278–U39. doi: 10.1136/heartjnl-2015-308403 26567228 10.1136/heartjnl-2015-308403 PMC4866903

[2] Knottnerus IL, Ten Cate H, Lodder J, Kessels F and van Oostenbrugge RJ. Endothelial dysfunction in lacunar stroke: a systematic review. *Cerebrovasc Dis*. 2009;27:519–26. doi: 10.1159/000212672 19372654 10.1159/000212672

[3] Santos M, Xekardaki A, Kovari E, Gold G, Bouras C and Giannakopoulos P. Microvascular pathology in late-life depression. *J Neurol Sci*. 2012;322:46–49. doi: 10.1016/j.jns.2012.05.048 22687957 10.1016/j.jns.2012.05.048

[4] De Silva TM and Faraci FM. Microvascular Dysfunction and Cognitive Impairment. *Cell Mol Neurobiol*. 2016;36:241–258. doi: 10.1007/s10571-015-0308-1 26988697 10.1007/s10571-015-0308-1 PMC4846472

[5] Gupta A and Bhatnagar S. Vasoregression: A Shared Vascular Pathology Underlying Macrovascular And Microvascular Pathologies? *OMICS*. 2015;19:733–53. doi: 10.1089/omi.2015.0128 26669709 10.1089/omi.2015.0128 PMC4684001

[6] Zafrani L and Ince C. Microcirculation in Acute and Chronic Kidney Diseases. *American Journal of Kidney Diseases*. 2015;66:1083–1094. doi: 10.1053/j.ajkd.2015.06.019 26231789 10.1053/j.ajkd.2015.06.019

[7] de Jongh RT, Serne EH, IJzerman RG, de Vries G and Stehouwer CD. Impaired microvascular function in obesity: implications for obesity-associated microangiopathy, hypertension, and insulin resistance. *Circulation*. 2004;109:2529–35. doi: 10.1161/01.CIR.0000129772.26647.6F 15136505 10.1161/01.CIR.0000129772.26647.6F

[8] Nagel E, Vilser W and Lanzl I. Age, blood pressure, and vessel diameter as factors influencing the arterial retinal flicker response. *Invest Ophthalmol Vis Sci*. 2004;45:1486–92. 15111606 10.1167/iovs.03-0667

[9] Reimann M, Weiss N and Ziemssen T. Different responses of the retinal and cutaneous microcirculation to transient dysmetabolic conditions. *Atheroscler Suppl*. 2015;18:1–7. doi: 10.1016/j.atherosclerosissup.2015.02.001 25936297 10.1016/j.atherosclerosissup.2015.02.001

[10] IJzerman RG, Serne EH, van Weissenbruch MM, de Jongh RT and Stehouwer CD. Cigarette smoking is associated with an acute impairment of microvascular function in humans. *Clin Sci (Lond)*. 2003;104:247–52. 12605581 10.1042/CS20020318

[11] IJzerman RG, de Jongh RT, Beijk MA, van Weissenbruch MM, Delemarre-van de Waal HA, Serne EH, et al Individuals at increased coronary heart disease risk are characterized by an impaired microvascular function in skin. *Eur J Clin Invest*. 2003;33:536–42. 12814388 10.1046/j.1365-2362.2003.01179.x

[12] Irving RJ, Walker BR, Noon JP, Watt GC, Webb DJ and Shore AC. Microvascular correlates of blood pressure, plasma glucose, and insulin resistance in health. *Cardiovasc Res*. 2002;53:271–6. 11744037 10.1016/s0008-6363(01)00450-3

[13] Caballero AE, Arora S, Saouaf R, Lim SC, Smakowski P, Park JY, et al Microvascular and macrovascular reactivity is reduced in subjects at risk for type 2 diabetes. *Diabetes*. 1999;48:1856–62. 10480619 10.2337/diabetes.48.9.1856

[14] Tsao CW and Vasan RS. Cohort Profile: The Framingham Heart Study (FHS): overview of milestones in cardiovascular epidemiology. *Int J Epidemiol*. 2015;44:1800–13. doi: 10.1093/ije/dyv337 26705418 10.1093/ije/dyv337 PMC5156338

[15] Chen SM, Tsai TH, Hang CL, Yip HK, Fang CY, Wu CJ, et al Endothelial dysfunction in young patients with acute ST-elevation myocardial infarction. *Heart Vessels*. 2011;26:2–9. doi: 10.1007/s00380-010-0017-0 20949355 10.1007/s00380-010-0017-0

[16] Witte DR, Westerink J, de Koning EJ, van der Graaf Y, Grobbee DE and Bots ML. Is the association between flow-mediated dilation and cardiovascular risk limited to low-risk populations? *J Am Coll Cardiol*. 2005;45:1987–93. doi: 10.1016/j.jacc.2005.02.073 15963397 10.1016/j.jacc.2005.02.073

[17] Houben A, Martens RJH and Stehouwer CDA. Assessing Microvascular Function in Humans from a Chronic Disease Perspective. *J Am Soc Nephrol*. 2017. 10.1681/ASN.2017020157 PMC5698072 28904002

[18] Minson CT, Berry LT and Joyner MJ. Nitric oxide and neurally mediated regulation of skin blood flow during local heating. *J Appl Physiol* (1985). 2001;91:1619–26. 11568143 10.1152/jappl.2001.91.4.1619

[19] Lim M, Sasongko MB, Ikram MK, Lamoureux E, Wang JJ, Wong TY, et al Systemic associations of dynamic retinal vessel analysis: a review of current literature. *Microcirculation*. 2013;20:257–68. doi: 10.1111/micc.12026 23151190 10.1111/micc.12026

[20] Lacolley P, Regnault V, Nicoletti A, Li Z and Michel JB. The vascular smooth muscle cell in arterial pathology: a cell that can take on multiple roles. *Cardiovasc Res*. 2012;95:194–204. doi: 10.1093/cvr/cvs135 22467316 10.1093/cvr/cvs135

[21] Montero D, Pierce GL, Stehouwer CD, Padilla J and Thijssen DH. The impact of age on vascular smooth muscle function in humans. *J Hypertens*. 2015;33:445–53; discussion 453. doi: 10.1097/HJH.0000000000000446 25479030 10.1097/HJH.0000000000000446 PMC4670263

[22] Aird WC. Endothelial cell heterogeneity. *Cold Spring Harb Perspect Med*. 2012;2:a006429 doi: 10.1101/cshperspect.a006429 22315715 10.1101/cshperspect.a006429 PMC3253027

[23] Fraser-Bell S, Symes R and Vaze A. Hypertensive eye disease: a review. *Clin Exp Ophthalmol*. 2017;45:45–53. doi: 10.1111/ceo.12905 27990740 10.1111/ceo.12905

[24] Buysschaert M, Medina JL, Bergman M, Shah A and Lonier J. Prediabetes and associated disorders. *Endocrine*. 2015;48:371–93. doi: 10.1007/s12020-014-0436-2 25294012 10.1007/s12020-014-0436-2

[25] Wickman C and Kramer H. Obesity and kidney disease: potential mechanisms. *Semin Nephrol*. 2013;33:14–22. doi: 10.1016/j.semnephrol.2012.12.006 23374890 10.1016/j.semnephrol.2012.12.006

[26] Parekh A, Smeeth D, Milner Y and Thure S. The Role of Lipid Biomarkers in Major Depression. *Healthcare (Basel)*. 2017;5. 10.3390/healthcare5010005 PMC5371911 28165367

[27] Kubli S, Waeber B, Dalle-Ave A and Feihl F. Reproducibility of laser Doppler imaging of skin blood flow as a tool to assess endothelial function. *J Cardiovasc Pharmacol*. 2000;36:640–8. 11065225 10.1097/00005344-200011000-00014

[28] Nagel E, Vilser W, Fink A and Riemer T. [Variance of retinal vessel diameter response to flicker light. A methodical clinical study]. *Ophthalmologe*. 2006;103:114–9. doi: 10.1007/s00347-005-1254-y 16170522 10.1007/s00347-005-1254-y

[29] Schram MT, Sep SJ, van der Kallen CJ, Dagnelie PC, Koster A, Schaper N, et al The Maastricht Study: an extensive phenotyping study on determinants of type 2 diabetes, its complications and its comorbidities. *Eur J Epidemiol*. 2014;29:439–51. doi: 10.1007/s10654-014-9889-0 24756374 10.1007/s10654-014-9889-0

[30] Sörensen BM, Houben AJ, Berendschot TT, Schouten JS, Kroon AA, van der Kallen CJ, et al Prediabetes and Type 2 Diabetes Are Associated With Generalized Microvascular Dysfunction: The Maastricht Study. *Circulation*. 2016;134:1339–1352. doi: 10.1161/CIRCULATIONAHA.116.023446 27678264 10.1161/CIRCULATIONAHA.116.023446

[31] Fitzmaurice G. The meaning and interpretation of interaction. *Nutrition*. 2000;16:313–4. 10758374 10.1016/s0899-9007(99)00293-2

[32] Puissant C, Abraham P, Durand S, Humeau-Heurtier A, Faure S, Rousseau P, et al [Endothelial function: role, assessment and limits]. *J Mal Vasc*. 2014;39:47–56. 24355615 10.1016/j.jmv.2013.11.004

[33] Jia G, Durante W and Sowers JR. Endothelium-Derived Hyperpolarizing Factors: A Potential Therapeutic Target for Vascular Dysfunction in Obesity and Insulin Resistance. *Diabetes*. 2016;65:2118–20. doi: 10.2337/dbi16-0026 27456617 10.2337/dbi16-0026 PMC4955984

[34] Kellogg DL Jr, Liu Y, Kosiba IF and O'Donnell D. Role of nitric oxide in the vascular effects of local warming of the skin in humans. *J Appl Physiol* (1985). 1999;86:1185–90. 10194201 10.1152/jappl.1999.86.4.1185

[35] Falsini B, Riva CE and Logean E. Flicker-evoked changes in human optic nerve blood flow: relationship with retinal neural activity. *Invest Ophthalmol Vis Sci*. 2002;43:2309–16. 12091432

[36] Seshadri S, Ekart A and Gherghel D. Ageing effect on flicker-induced diameter changes in retinal microvessels of healthy individuals. *Acta Ophthalmol*. 2016;94:e35–42. doi: 10.1111/aos.12786 26149453 10.1111/aos.12786 PMC5034828

[37] Ogueta SB, Schwartz SD, Yamashita CK and Farber DB. Estrogen receptor in the human eye: influence of gender and age on gene expression. *Invest Ophthalmol Vis Sci*. 1999;40:1906–11. 10440242

[38] Riva CE, Logean E and Falsini B. Visually evoked hemodynamical response and assessment of neurovascular coupling in the optic nerve and retina. *Prog Retin Eye Res*. 2005;24:183–215. doi: 10.1016/j.preteyeres.2004.07.002 15610973 10.1016/j.preteyeres.2004.07.002

[39] Yang SH, Liu R, Perez EJ, Wang X and Simpkins JW. Estrogens as protectants of the neurovascular unit against ischemic stroke. *Curr Drug Targets CNS Neurol Disord*. 2005;4:169–77. 15857302 10.2174/1568007053544174

[40] Celermajer DS, Sorensen KE, Spiegelhalter DJ, Georgakopoulos D, Robinson J and Deanfield JE. Aging is associated with endothelial dysfunction in healthy men years before the age-related decline in women. *J Am Coll Cardiol*. 1994;24:471–6. 8034885 10.1016/0735-1097(94)90305-0

[41] Cracowski JL. Female hormones and skin microvascular function. *Microcirculation*. 2011;18:356–7. doi: 10.1111/j.1549-8719.2011.00098.x 21418387 10.1111/j.1549-8719.2011.00098.x

[42] Arora S, Veves A, Caballaro AE, Smakowski P and LoGerfo FW. Estrogen improves endothelial function. *J Vasc Surg*. 1998;27:1141–1146. 9652476 10.1016/s0741-5214(98)70016-3

[43] Smith AR, Visioli F and Hagen TM. Plasma membrane-associated endothelial nitric oxide synthase and activity in aging rat aortic vascular endothelia markedly decline with age. *Arch Biochem Biophys*. 2006;454:100–105. doi: 10.1016/j.abb.2006.02.017 16982030 10.1016/j.abb.2006.02.017

[44] Chilelli NC, Burlina S and Lapolla A. AGEs, rather than hyperglycemia, are responsible for microvascular complications in diabetes: a "glycoxidation-centric" point of view. *Nutr Metab Cardiovasc Dis*. 2013;23:913–9. doi: 10.1016/j.numecd.2013.04.004 23786818 10.1016/j.numecd.2013.04.004

[45] Jonk AM, Houben AJ, de Jongh RT, Serne EH, Schaper NC and Stehouwer CD. Microvascular dysfunction in obesity: a potential mechanism in the pathogenesis of obesity-associated insulin resistance and hypertension. *Physiology (Bethesda)*. 2007;22:252–60. 17699878 10.1152/physiol.00012.2007

[46] Hashimoto S, Kubota N, Sato H, Sasaki M, Takamoto I, Kubota T, et al Insulin receptor substrate-2 (Irs2) in endothelial cells plays a crucial role in insulin secretion. *Diabetes*. 2015;64:876–86. doi: 10.2337/db14-0432 25277391 10.2337/db14-0432

[47] Cheung N, Sharrett AR, Klein R, Criqui MH, Islam FM, Macura KJ, et al Aortic distensibility and retinal arteriolar narrowing: the multi-ethnic study of atherosclerosis. *Hypertension*. 2007;50:617–22. doi: 10.1161/HYPERTENSIONAHA.107.091926 17698721 10.1161/HYPERTENSIONAHA.107.091926

[48] Henriksson P, Lu Q, Diczfalusy U and Freyschuss A. Immediate effect of passive smoking on microcirculatory flow. *Microcirculation*. 2014;21:587–92. doi: 10.1111/micc.12137 24698527 10.1111/micc.12137

[49] Garhofer G, Resch H, Sacu S, Weigert G, Schmidl D, Lasta M, et al Effect of regular smoking on flicker induced retinal vasodilatation in healthy subjects. *Microvasc Res*. 2011;82:351–5. doi: 10.1016/j.mvr.2011.07.001 21771603 10.1016/j.mvr.2011.07.001

[50] Muris DM, Houben AJ, Kroon AA, Henry RM, van der Kallen CJ, Sep SJ, et al Age, waist circumference, and blood pressure are associated with skin microvascular flow motion: the Maastricht Study. *J Hypertens*. 2014;32:2439–49; discussion 2449. 25222377 10.1097/HJH.0000000000000348

[51] Francischetti EA, Tibirica E, da Silva EG, Rodrigues E, Celoria BM and de Abreu VG. Skin capillary density and microvascular reactivity in obese subjects with and without metabolic syndrome. *Microvasc Res*. 2011;81:325–30. doi: 10.1016/j.mvr.2011.01.002 21236266 10.1016/j.mvr.2011.01.002

[52] Yudkin JS, Eringa E and Stehouwer CD. "Vasocrine" signalling from perivascular fat: a mechanism linking insulin resistance to vascular disease. *Lancet*. 2005;365:1817–20. doi: 10.1016/S0140-6736(05)66585-3 15910955 10.1016/S0140-6736(05)66585-3

[53] Seshadri S, Mroczkowska S, Qin L, Patel S, Ekart A and Gherghel D. Systemic circulatory influences on retinal microvascular function in middle-age individuals with low to moderate cardiovascular risk. *Acta ophthalmologica*. 2015;93:e266–74. doi: 10.1111/aos.12594 25487686 10.1111/aos.12594

[54] Muris DM, Houben AJ, Schram MT and Stehouwer CD. Microvascular dysfunction: an emerging pathway in the pathogenesis of obesity-related insulin resistance. *Rev Endocr Metab Disord*. 2013;14:29–38. doi: 10.1007/s11154-012-9231-7 23299657 10.1007/s11154-012-9231-7

