# Supplementary material for: Cardiovascular risk factors as determinants of retinal and skin microvascular function: The Maastricht Study
Source: PLoS One. 2017 Oct 27;12(10):e0187324. doi: 10.1371/journal.pone.0187324 (PMC5659678; doi:10.1371/journal.pone.0187324)
Supplement: S1 Appendix — (DOCX) [file pone.0187324.s001.docx]

**S1 Appendix: Assessment of retinal and skin microvascular function.**

*Retinal arteriolar dilation response* [1]

For retinal measurements pupils were dilated with 0.5% tropicamide and 2.5% phenylephrine at least 15 minutes prior to the start of the examination. The retinal arteriolar dilation response to flicker light was measured in a dimly lit room by use of the Dynamic Vessel Analyzer (DVA) (Imedos, Jena, Germany). For safety reasons, participants with an intraocular pressure exceeding 30 mmHg were excluded from retinal measurements. Per participant, we randomly measured the left or right eye.

During the measurement, the participant was instructed and encouraged to focus on the tip of a fixated needle inside the retinal camera (FF450; Carl Zeiss GmbH, Jena, Germany), while the fundus of the eye was examined under green measuring light (530-600 nm, illumination of fundus approximately 6500 lux). A straight arteriolar segment of approximately 1.5 mm in length located 0.5 to 2.0 disc diameter from the margin of the optic disc in the temporal section was examined. When the specific vessel profile was recognized, vessel diameter was automatically and continuously measured for 150 seconds. A baseline recording of 50 seconds was followed by a 40-second flicker light exposure period (flicker frequency 12.5Hz, bright-to-dark contrast ratio 25:1) followed by a 60-second recovery period. The DVA automatically corrected for alterations in luminance caused by, for example, slight eye movements. During blinks and small eye movements, the registration stopped and restarted once the vessel segments were automatically re-identified.

The integrated DVA software (version 4.51, Imedos) automatically calculated baseline diameter and percentage dilation. Baseline diameter was calculated as the average diameter size of the 20-50 seconds recording and was expressed in measurement units (MU), where 1 MU is equal to 1µm of the Gullstrand eye [2]. Percentage dilation over baseline was based on the average dilation achieved at time points 10 and 40 seconds during the flicker stimulation period. The purpose of taking the average dilation was to account for inter-individual variation in the curve shape during dilation.

*Skin hyperemic response* [1]

Skin blood flow measurements were performed in a climate-controlled room at 24°C with participants in a supine position. Skin blood flow was measured as described previously by means of a laser-Doppler system (Periflux 5000, Perimed, Järfälla, Sweden), equipped with a thermostatic laser-Doppler probe (PF457) at the dorsal side of the wrist of the left hand [3]. The laser-Doppler output was recorded for 25 minutes with a sample rate of 32Hz, which gives semi-quantitative assessment of skin blood flow expressed in arbitrary perfusion units (PU). Skin blood flow was first recorded unheated for 2 minutes to serve as a baseline. After the 2 minutes of baseline, the temperature of the probe was rapidly and locally increased to 44°C, and was then kept constant until the end of the registration. The heat-induced skin hyperemic response was expressed as the percentage increase in average PU during the 23 minutes heating phase over the average baseline PU.

**References**

1. Sörensen BM, Houben AJ, Berendschot TT, Schouten JS, Kroon AA, van der Kallen CJ, et al. Prediabetes and type 2 diabetes are associated with generalized microvascular dysfunction: The Maastricht Study. *Circulation*. 2016;134:1339-1352.
2. Nagel E, Vilser W, Fink A and Riemer T. [Variance of retinal vessel diameter response to flicker light. A methodical clinical study]. *Ophthalmologe*. 2006;103:114-9.
3. Muris DM, Houben AJ, Kroon AA, Henry RM, van der Kallen CJ, Sep SJ, et al. Age, waist circumference, and blood pressure are associated with skin microvascular flow motion: the Maastricht Study. *J Hypertens*. 2014;32:2439-49; discussion 2449.
