# Supplementary material for: Cardiovascular risk factors as determinants of retinal and skin microvascular function: The Maastricht Study
Source: PLoS One. 2017 Oct 27;12(10):e0187324. doi: 10.1371/journal.pone.0187324 (PMC5659678; doi:10.1371/journal.pone.0187324)
Supplement: S2 Appendix — (DOCX) [file pone.0187324.s002.docx]

**S2 Appendix: Details on the assessment of cardiovascular risk factors.**

*Glucose metabolism status*

To assess glucose metabolism status, all participants (except those who used insulin) underwent a standardized 2-h 75 gram oral glucose tolerance test (OGTT) after an overnight fast. For safety reasons, participants with a fasting glucose level above 11.0 mmol/l, as determined by a finger prick, did not undergo the OGTT. For these individuals fasting glucose level and information about diabetes medication use were used to assess glucose metabolism status. Glucose metabolism status was defined according to the World Health Organization 2006 criteria as normal glucose metabolism (NGM), impaired fasting glucose, impaired glucose tolerance (combined as prediabetes) and type 2 diabetes. Additionally, individuals without type 1 diabetes and no diabetes medication were considered as having type 2 diabetes [1].

*Measures of glycemia*

Venous fasting and postload plasma glucose levels were measured by the enzymatic hexokinase method on two automatic analyzers (i.e. the Beckman Synchron LX20 (Beckman Coulter Inc., USA) for samples obtained between November 2010 and April 2012, and the Roche Cobas 6000 (Roche Diagnostics, Mannheim, Germany) for samples obtained thereafter). Glycated hemoglobin A1c (HbA1c) was determined by ion-exchange high performance liquid chromatography [1].

*Measures of blood pressure*

Participants underwent 24-hs blood pressure monitoring (WatchBP03, Microlife AG, Widnau, Switzerland) with an appropriate sized cuff placed around their non-dominant arm [1]. The device was programmed to take blood pressure readings every 15 minutes during daytime (8 a.m. – 11 p.m.) and every 30 minutes during the night (11 p.m. – 8 a.m.). According to recommendations of the British Hypertension society [2], mean 24-h blood pressure measurements were calculated only if there were more than 14 valid blood pressure measurements during daytime and more than 7 valid measurements during nighttime. 24-h mean arterial pressure was calculated as 24-h diastolic blood pressure (DBP) + (1/3 * (24-h systolic blood pressure (SBP) – 24-h DBP)). 24-h pulse pressure was calculated as 24-h SBP – 24-h DBP. The use of antihypertensive medication was assessed during a medication interview where generic name, dose, and frequency were registered [1].

*Measures of lipid profile*

Serum concentrations of total cholesterol, high-density lipoprotein (HDL) cholesterol and triglycerides were measured using an automatic analyzer (Beckman Synchron LX20, Beckman Coulter Inc. Brea, USA). The use of lipid-modifying medication was assessed during a medication interview where generic name, dose, and frequency were registered [1].

**References**

1. Schram MT, Sep SJ, van der Kallen CJ, Dagnelie PC, Koster A, Schaper N, et al. The Maastricht Study: an extensive phenotyping study on determinants of type 2 diabetes, its complications and its comorbidities. *Eur J Epidemiol*. 2014;29:439-51.
2. O'Brien E, Coats A, Owens P, Petrie J, Padfield PL, Littler WA, et al. Use and interpretation of ambulatory blood pressure monitoring: recommendations of the British hypertension society. *BMJ*. 2000;320:1128-34.
