# Supplementary material for: Cardiovascular risk factors as determinants of retinal and skin microvascular function: The Maastricht Study
Source: PLoS One. 2017 Oct 27;12(10):e0187324. doi: 10.1371/journal.pone.0187324 (PMC5659678; doi:10.1371/journal.pone.0187324)
Supplement: S3 Appendix — (DOCX) [file pone.0187324.s003.docx]

**S3 Details on the assessment of covariates.**

We measured height, weight, office blood pressure, serum creatinine and cystatin C [1], and
24-h urinary albumin excretion (twice) as described previously [2]. Body mass index was calculated as weight (kg) divided by height (m^2^). Estimated glomerular filtration rate (eGFR; in ml/min/1.73m^2^) was calculated with the Chronic Kidney Disease Epidemiology Collaboration (CDK-epi) equation based on both serum creatinine and serum cystatin C. The presence of retinopathy was based on fundus photographs taken with an auto fundus camera (Model AFC-230, Nidek, Gamagori, Japan). A history of cardiovascular disease (CVD) was assessed by web-based questionnaires and was defined as a history of myocardial infarction, stroke or vascular surgery (including angioplasty) of coronary, carotid, abdominal aortic or peripheral arteries [2].

**References**

1. Martens RJ, Henry RM, Houben AJ, van der Kallen CJ, Kroon AA, Schalkwijk CG, et al. Capillary rarefaction associates with albuminuria: The Maastricht Study. *J Am Soc Nephrol*. 2016;27:3748-3757.
2. Schram MT, Sep SJ, van der Kallen CJ, Dagnelie PC, Koster A, Schaper N, et al. The Maastricht Study: an extensive phenotyping study on determinants of type 2 diabetes, its complications and its comorbidities. *Eur J Epidemiol*. 2014;29:439-51.
