# Supplementary material for: Cardiovascular risk factors as determinants of retinal and skin microvascular function: The Maastricht Study
Source: PLoS One. 2017 Oct 27;12(10):e0187324. doi: 10.1371/journal.pone.0187324 (PMC5659678; doi:10.1371/journal.pone.0187324)
Supplement: S1 Table — Data are reported as mean ± SD, median [interquartile range], or number (percentages %) as appropriate. P-value indicates comparison between study population and individuals excluded due to missing values. SD, standard deviation; SBP, systolic blood pressure; DBP, diastolic blood pressure; PP, pulse pressure; MAP, mean arterial pressure; HbA1c, glycated hemoglobin A1c; HDL, high-density lipoprotein; LDL, low-density lipoprotein; eGFR, estimated glomerular filtration rate; MU, measurement units. * = Total number of missing values for a specific variable in the retinal reactivity study population, † = Total number of missing values for a specific variable in the population which was excluded, ‡ = (Micro)albuminuria was defined as a urinary albumin excretion of >30 mg per 24 hours, § = 299 were excluded due to missing on potential cardiovascular risk factors. (DOCX) [file pone.0187324.s004.docx]

**S1 Table**: General characteristics for the retinal reactivity study population and individuals excluded from the analyses due to missing values

| **Characteristic** | **Retinal reactivity study population (n=1991)** | **Missings in retinal reactivity study population*** | **Excluded due to missing values (n=1460)** | **Missings in population excluded due to missing values**^†^ | **P-value** |
| --- | --- | --- | --- | --- | --- |
| Age (years) | 59.7+8.2 | 0 | 59.8+8.3 | 0 | 0.858 |
| Women | 971 (48.8) | 0 | 705 (48.3) | 0 | 0.780 |
| - Postmenopausal | 726 (78.1) | 42 | 482 (77.0) | 79 | 0.593 |
| - Hormone replacement therapy | 17 (1.8) | 0 | 18 (1.2) | 4 | 0.269 |
| Glucose metabolism status |  | 0 |  | 0 | 0.506 |
| - Normal glucose metabolism | 1131 (56.8) |  | 793 (54.3) |  |  |
| - Prediabetes | 292 (14.7) |  | 219 (15.0) |  |  |
| - Type 2 diabetes | 545 (27.4) |  | 430 (29.5) |  |  |
| - Other types of diabetes | 23 (1.2) |  | 18 (1.2) |  |  |
| Type 2 diabetes duration (years) | 6.0 [3.0-12.0] | 167 | 7.0 [3.0-12.0] | 144 | 0.307 |
| Body mass index (kg/m^2^) | 26.9+4.4 | 1 | 27.4+4.7 | 2 | 0.001 |
| Weight (kg) | 78.9+15.7 | 0 | 80.3+15.8 | 2 | 0.012 |
| Height (cm) | 171.1+8.8 | 1 | 171.0+9.1 | 2 | 0.906 |
| Waist circumference (cm) |  | 0 |  | 4 |  |
| - Men | 101.1+11.8 |  | 102.1+12.5 |  | 0.092 |
| - Women | 89.0+12.5 |  | 91.4+13.5 |  | <0.001 |
| History of cardiovascular disease | 306 (15.6) |  | 252 (18.3) |  | 0.040 |
| Office SBP (mmHg) | 135.0+18.0 | 2 | 135.2+18.5 | 0 | 0.756 |
| Office DBP (mmHg) | 76.3+9.9 | 2 | 76.0+9.8 | 0 | 0.337 |
| Ambulatory 24-h SBP (mmHg) | 119.1+11.6 | 0 | 119.0+12.3 | 404 | 0.669 |
| Ambulatory 24-h DBP (mmHg) | 73.5+7.2 | 0 | 73.5+7.2 | 404 | 0.923 |
| Ambulatory 24-h PP (mmHg) | 45.6+8.5 | 0 | 45.5+9.1 | 404 | 0.617 |
| Ambulatory 24-h MAP (mmHg) | 88.7+7.9 | 0 | 88.6+8.1 | 404 | 0.788 |
| Smoking |  | 0 |  | 63 | 0.002 |
| - Never / former / current | 699/1051/241 |  | 471/698/228 |  |  |
| - % (never / former / current) | 35.1/52.8/12.1 |  | 33.7/50.0/16.3 |  |  |
| Pack-years of smoking | 1.8 [0.0-16.5] | 319 | 3.9 [0.0-20.0] | 235 | 0.003 |
| Fasting glucose (mmol/l) | 6.1+1.7 | 0 | 6.2+1.9 | 1 | 0.051 |
| 2-h postload glucose (mmol/l) | 7.9+4.2 | 149 | 7.9+4.3 | 143 | 0.818 |
| HbA1c (%) | 5.9+0.9 | 3 | 6.0+1.0 | 10 | <0.001 |
| HbA1c (mmol/mol) | 40.8+10.1 | 3 | 42.1+10.5 | 10 | <0.001 |
| Total-to-HDL cholesterol ratio | 3.6+1.1 | 0 | 3.8+1.2 | 4 | <0.001 |
| Total cholesterol (mmol/l) | 5.2+1.2 | 0 | 5.2+1.1 | 4 | 0.196 |
| HDL cholesterol (mmol/l) | 1.6+0.5 | 0 | 1.5+0.5 | 4 | <0.001 |
| LDL cholesterol (mmol/l) | 3.1+1.0 | 0 | 3.1+1.0 | 4 | 0.412 |
| Triglycerides (mmol/l) | 1.4+0.8 | 0 | 1.4+0.9 | 4 | 0.295 |
| Antihypertensive medication use | 760 (38.2) | 0 | 618 (42.4) | 4 | 0.011 |
| Lipid-modifying medication use | 706 (35.5) | 0 | 542 (37.2) | 4 | 0.287 |
| Diabetes medication use |  |  |  |  |  |
| - Any type | 446 (22.4) | 0 | 360 (24.7) | 4 | 0.111 |
| - Insulin | 132 (6.6) | 0 | 125 (8.6) | 4 | 0.031 |
| - Oral glucose-lowering medication | 398 (20.0) | 0 | 318 (21.8) | 4 | 0.186 |
| eGFR (ml/min/1.73m^2^) | 88.2+14.6 | 13 | 88.0+15.4 | 20 | 0.664 |
| eGFR<60 ml/min/1.73m^2^ | 85 (4.3) | 13 | 62 (4.3) | 20 | 0.991 |
| (Micro)albuminuria^‡^ | 163 (8.2) | 12 | 136 (9.5) | 30 | 0.194 |
| Retinopathy | 32 (1.7) | 56 | 14 (1.5) | 538 | 0.788 |
| Baseline arteriolar diameter (MU) | 115.4+15.6 | 0 | 115.8+16.2^§^ | 1161 | 0.670 |
| Arteriolar average dilation (%) |  |  |  |  |  |
| - Mean + SD | 3.0+2.8 | 0 | 2.9+2.7^§^ | 1161 | 0.526 |
| - Median (interquartile range) | 2.6 [0.8-4.9] | 0 | 2.5 [0.8-4.9]^§^ | 1161 | 0.526 |

Data are reported as mean ± SD, median [interquartile range], or number (percentages %) as appropriate. P-value indicates comparison between study population and individuals excluded due to missing values. SD, standard deviation; SBP, systolic blood pressure; DBP, diastolic blood pressure; PP, pulse pressure; MAP, mean arterial pressure; HbA1c, glycated hemoglobin A1c; HDL, high-density lipoprotein; LDL, low-density lipoprotein; eGFR, estimated glomerular filtration rate; MU, measurement units. *=Total number of missing values for a specific variable in the retinal reactivity study population, †=Total number of missing values for a specific variable in the population which was excluded, ‡=(Micro)albuminuria was defined as a urinary albumin excretion of >30 mg per 24 hours, §=299 were excluded due to missing on potential cardiovascular risk factors.
