# Supplementary material for: Cardiovascular risk factors as determinants of retinal and skin microvascular function: The Maastricht Study
Source: PLoS One. 2017 Oct 27;12(10):e0187324. doi: 10.1371/journal.pone.0187324 (PMC5659678; doi:10.1371/journal.pone.0187324)
Supplement: S2 Table — Data are reported as mean ± SD, median [interquartile range], or number (percentages %) as appropriate. P-value indicates comparison between study population and individuals excluded due to missing values. SD, standard deviation; SBP, systolic blood pressure; DBP, diastolic blood pressure; PP, pulse pressure; MAP, mean arterial pressure; HbA1c, glycated hemoglobin A1c; HDL, high-density lipoprotein; LDL, low-density lipoprotein; eGFR, estimated glomerular filtration rate; PU, perfusion units. * = Total number of missing values for a specific variable in the skin hyperemia study population, † = Total number of missing values for a specific variable in the population which was excluded, ‡ = (Micro)albuminuria was defined as a urinary albumin excretion of >30 mg per 24 hours, § = 249 were excluded due to missing on potential cardiovascular risk factors. (DOCX) [file pone.0187324.s005.docx]

**Supplemental Table S2**: General characteristics for the skin hyperemia study population and individuals excluded from the analyses due to missing values

| **Characteristic** | **Skin hyperemia  study population  (n=1427)** | **Missings in skin hyperemia study population*** | **Excluded due to missing values (n=2024)** | **Missings in population excluded due to missing values**^†^ | **P-value** |
| --- | --- | --- | --- | --- | --- |
| Age (years) | 60.2+8.1 | 0 | 59.5+8.4 | 0 | 0.010 |
| Women | 674 (47.2) | 0 | 1002 (49.5) | 0 | 0.188 |
| - Postmenopausal | 478 (77.5) | 57 | 730 (77.8) | 64 | 0.870 |
| - Hormone replacement therapy | 15 (2.2) | 0 | 20 (2.0) | 4 | 0.860 |
| Glucose metabolism status |  | 0 |  | 0 | 0.073 |
| - Normal glucose metabolism | 763 (53.5) |  | 1161 (57.4) |  |  |
| - Prediabetes | 222 (15.6) |  | 289 (14.3) |  |  |
| - Type 2 diabetes | 420 (29.4) |  | 555 (27.4) |  |  |
| - Other types of diabetes | 22 (1.5) |  | 19 (0.9) |  |  |
| Type 2 diabetes duration (years) | 6.0 [3.0-11.0] | 119 | 7.0 [3.0-13.0] | 192 | 0.104 |
| Body mass index (kg/m^2^) | 26.9+4.4 | 1 | 27.2+4.7 | 2 | 0.088 |
| Weight (kg) | 79.1+15.0 | 0 | 79.8+16.2 | 2 | 0.186 |
| Height (cm) | 171.1+8.7 | 1 | 171.0+9.1 | 2 | 0.683 |
| Waist circumference (cm) |  | 0 |  | 4 |  |
| - Men | 101.0+11.6 |  | 101.9+12.4 |  | 0.134 |
| - Women | 90.1+12.8 |  | 90.0+13.2 |  | 0.874 |
| History of cardiovascular disease | 249 (17.7) | 19 | 309 (16.0) | 89 | 0.189 |
| Office SBP (mmHg) | 135.8+18.2 | 1 | 134.6+18.2 | 1 | 0.056 |
| Office DBP (mmHg) | 76.5+9.6 | 1 | 75.9+10.0 | 1 | 0.116 |
| Ambulatory 24-h SBP (mmHg) | 119.8+11.7 | 0 | 118.5+11.9 | 404 | 0.002 |
| Ambulatory 24-h DBP (mmHg) | 73.6+6.9 | 0 | 73.4+7.4 | 404 | 0.322 |
| Ambulatory 24-h PP (mmHg) | 46.2+8.9 | 0 | 45.1+8.6 | 404 | 0.001 |
| Ambulatory 24-h MAP (mmHg) | 89.0+7.7 | 0 | 88.4+8.2 | 404 | 0.034 |
| Smoking |  | 0 |  | 61 | 0.006 |
| - Never / former / current | 469/781/177 |  | 701/968/292 |  |  |
| - % (never / former / current) | 32.9/54.7/12.4 |  | 35.7/49.4/14.9 |  |  |
| Pack-years of smoking | 1.7 [0.0-17.0] | 196 | 4.4 [0.0-20.0] | 358 | 0.007 |
| Fasting glucose (mmol/l) | 6.1+1.6 | 0 | 6.1+1.9 | 1 | 0.890 |
| 2-h postload glucose (mmol/l) | 8.1+4.4 | 116 | 7.8+4.2 | 176 | 0.030 |
| HbA1c (%) | 6.0+0.9 | 1 | 5.9+1.0 | 12 | 0.053 |
| HbA1c (mmol/mol) | 41.8+10.1 | 1 | 41.1+10.4 | 12 | 0.050 |
| Total-to-HDL cholesterol ratio | 3.7+1.1 | 0 | 3.7+1.2 | 4 | 0.623 |
| Total cholesterol (mmol/l) | 5.2+1.2 | 0 | 5.2+1.1 | 4 | 0.644 |
| HDL cholesterol (mmol/l) | 1.5+0.5 | 0 | 1.5+0.5 | 4 | 0.454 |
| LDL cholesterol (mmol/l) | 3.1+1.1 | 0 | 3.1+1.0 | 4 | 0.407 |
| Triglycerides (mmol/l) | 1.4+0.9 | 0 | 1.4+0.8 | 4 | 0.302 |
| Antihypertensive medication use | 593 (41.6) | 0 | 785 (38.9) | 4 | 0.112 |
| Lipid-modifying medication use | 557 (39.0) | 0 | 691 (34.2) | 4 | 0.004 |
| Diabetes medication use |  |  |  |  |  |
| - Any type | 351 (24.6) | 0 | 455 (22.5) | 4 | 0.157 |
| - Insulin | 105 (7.4) | 0 | 152 (7.5) | 4 | 0.854 |
| - Oral glucose-lowering medication | 311 (21.8) | 0 | 405 (20.0) | 4 | 0.214 |
| eGFR (ml/min/1.73m^2^) | 88.3+14.5 | 11 | 88.0+15.3 | 22 | 0.455 |
| eGFR<60 ml/min/1.73m^2^ | 51 (3.6) | 11 | 96 (4.8) | 22 | 0.090 |
| (Micro)albuminuria^‡^ | 117 (8.2) | 7 | 182 (9.2) | 35 | 0.354 |
| Retinopathy | 21 (1.6) | 140 | 25 (1.6) | 454 | 0.934 |
| Baseline skin blood flow (PU) | 11.1+6.5 | 0 | 11.2+5.6^§^ | 1775 | 0.724 |
| Skin hyperemic response (%) |  |  |  |  |  |
| - Mean + SD | 1129.5+778.2 | 0 | 1069.4+713.0^§^ | 1775 | 0.255 |
| - Median (interquartile range) | 1002.6 [587.2-1508.9] | 0 | 915.0 [560.6-1415.4]^§^ | 1775 | 0.255 |

Data are reported as mean ± SD, median [interquartile range], or number (percentages %) as appropriate. P-value indicates comparison between study population and individuals excluded due to missing values. SD, standard deviation; SBP, systolic blood pressure; DBP, diastolic blood pressure; PP, pulse pressure; MAP, mean arterial pressure; HbA1c, glycated hemoglobin A1c; HDL, high-density lipoprotein; LDL, low-density lipoprotein; eGFR, estimated glomerular filtration rate; PU, perfusion units. *=Total number of missing values for a specific variable in the skin hyperemia study population, †=Total number of missing values for a specific variable in the population which was excluded, ‡=(Micro)albuminuria was defined as a urinary albumin excretion of >30 mg per 24 hours, §=249 were excluded due to missing on potential cardiovascular risk factors.
