# Supplementary material for: Cardiovascular risk factors as determinants of retinal and skin microvascular function: The Maastricht Study
Source: PLoS One. 2017 Oct 27;12(10):e0187324. doi: 10.1371/journal.pone.0187324 (PMC5659678; doi:10.1371/journal.pone.0187324)
Supplement: S4 Table — Point estimates (standardized beta) and 95%CIs represent the difference (in SD) in skin %-hyperemia per SD increase in the cardiovascular risk factor, men versus women, current smoker versus never smoker, or the use of antihypertensive or lipid-modifying medication versus no use. All associations were adjusted for the other risk factors with multivariate regression. Associations of sex were additionally adjusted for height. Note that as a consequence of standardization of the continuous variables (age, waist circumference, fasting plasma glucose, total-to-HDL cholesterol, 24-h systolic blood pressure) the regression coefficient (B) for continuous variables equals the standardized beta. *P<0.05, SD, standard deviation; CI, confidence interval; HDL, high-density lipoprotein. (DOCX) [file pone.0187324.s007.docx]

**Supplemental Table S4**: Multivariable-adjusted regression analyses of associations between cardiovascular risk factors with skin %-hyperemia

|  | Unstandardized coefficients | Standardized coefficients |  | 95% confidence interval for B | |
| --- | --- | --- | --- | --- | --- |
| **Determinant** | **B** | **Standardized beta** | **P-value** | **Lower bound** | **Upper bound** |
| Age* | -0.10 | -0.10 | <0.001 | -0.16 | -0.05 |
| Sex (men) * | -0.43 | -0.21 | <0.001 | -0.58 | -0.27 |
| Waist circumference | 0.05 | 0.05 | 0.115 | -0.01 | 0.12 |
| Fasting plasma glucose* | -0.11 | -0.11 | <0.001 | -0.17 | -0.06 |
| Total:HDL cholesterol ratio | 0.03 | 0.03 | 0.361 | -0.03 | 0.08 |
| 24-h systolic blood pressure | -0.04 | -0.04 | 0.200 | -0.09 | 0.02 |
| Smoking (current) * | -0.32 | -0.11 | <0.001 | -0.49 | -0.15 |
| Use of lipid-modifying drugs | -0.03 | -0.01 | 0.697 | -0.15 | 0.10 |
| Use of antihypertensive drugs | -0.05 | -0.03 | 0.396 | -0.17 | 0.07 |

Point estimates (standardized beta) and 95%CIs represent the difference (in SD) in skin %-hyperemia per SD increase in the cardiovascular risk factor, men versus women, current smoker versus never smoker, or the use of antihypertensive or lipid-modifying medication versus no use. All associations were adjusted for the other risk factors with multivariate regression. Associations of sex were additionally adjusted for height. Note that as a consequence of standardization of the continuous variables (age, waist circumference, fasting plasma glucose, total-to-HDL cholesterol, 24-h systolic blood pressure) the regression coefficient (B) for continuous variables equals the standardized beta. *P<0.05, SD, standard deviation; CI, confidence interval; HDL, high-density lipoprotein.
